# Age of first exposure to American football and long-term neuropsychiatric and cognitive outcomes

**DOI:** 10.1038/tp.2017.197

**Published:** 2017-09-19

**Authors:** M L Alosco, A B Kasimis, J M Stamm, A S Chua, C M Baugh, D H Daneshvar, C A Robbins, M Mariani, J Hayden, S Conneely, R Au, A Torres, M D McClean, A C McKee, R C Cantu, J Mez, C J Nowinski, B M Martin, C E Chaisson, Y Tripodis, R A Stern

**Affiliations:** 1 Boston University Alzheimer’s Disease and CTE Center, Boston University School of Medicine, Boston, MA, USA; 2 Department of Neurology, Boston University School of Medicine, Boston, MA, USA; 3 Department of Kinesiology, University of Wisconsin-Madison, Madison, WI, USA; 4 Department of Biostatistics, Boston University School of Public Health, Boston, MA, USA; 5 Interfaculty Initiative in Health Policy, Harvard University, Boston, MA, USA; 6 Concussion Legacy Foundation, Boston, MA, USA; 7 Boston University School of Medicine, Boston, MA, USA; 8 Department of Epidemiology, Boston University School of Public Health, Boston, MA, USA; 9 Department of Anatomy and Neurobiology, Boston University School of Medicine, Boston, MA, USA; 10 Pediatric Neurology, Boston University School of Medicine, Boston, MA, USA; 11 Pediatric Neurology, Boston Medical Center, Boston, MA, USA; 12 Department of Environmental Health, Boston University School of Public Health, Boston, MA, USA; 13 VA Boston Healthcare System, U.S. Department of Veteran Affairs, Boston, MA, USA; 14 Department of Pathology and Laboratory Medicine, Boston University School of Medicine, Boston, MA, USA; 15 Department of Veterans Affairs Medical Center, Bedford, MA, USA; 16 Department of Neurosurgery, Boston University School of Medicine, Boston, MA, USA; 17 Department of Neurosurgery, Emerson Hospital, Concord, MA, USA; 18 Data Coordinating Center, Boston University School of Public Health, Boston, MA, USA

## Abstract

Previous research suggests that age of first exposure (AFE) to football before age 12 may have long-term clinical implications; however, this relationship has only been examined in small samples of former professional football players. We examined the association between AFE to football and behavior, mood and cognition in a large cohort of former amateur and professional football players. The sample included 214 former football players without other contact sport history. Participants completed the Brief Test of Adult Cognition by Telephone (BTACT), and self-reported measures of executive function and behavioral regulation (Behavior Rating Inventory of Executive Function-Adult Version Metacognition Index (MI), Behavioral Regulation Index (BRI)), depression (Center for Epidemiologic Studies Depression Scale (CES-D)) and apathy (Apathy Evaluation Scale (AES)). Outcomes were continuous and dichotomized as clinically impaired. AFE was dichotomized into <12 and ⩾12, and examined continuously. Multivariate mixed-effect regressions controlling for age, education and duration of play showed AFE to football before age 12 corresponded with >2 × increased odds for clinically impaired scores on all measures but BTACT: (odds ratio (OR), 95% confidence interval (CI): BRI, 2.16,1.19–3.91; MI, 2.10,1.17–3.76; CES-D, 3.08,1.65–5.76; AES, 2.39,1.32–4.32). Younger AFE predicted increased odds for clinical impairment on the AES (OR, 95% CI: 0.86, 0.76–0.97) and CES-D (OR, 95% CI: 0.85, 0.74–0.97). There was no interaction between AFE and highest level of play. Younger AFE to football, before age 12 in particular, was associated with increased odds for impairment in self-reported neuropsychiatric and executive function in 214 former American football players. Longitudinal studies will inform youth football policy and safety decisions.

## Introduction

Exposure to repetitive head impacts (RHI) during American football has become a significant concern to clinicians, researchers and the general community because of their association with long-term neurological consequences.^1^ RHI exposure, with or without symptomatic concussions, can alter the structure and function of the brain to potentially underpin cognitive, behavior and mood deficits observed in some former amateur and professional football players.^1, 2, 3, 4, 5, 6, 7, 8, 9, 10, 11, 12, 13, 14, 15, 16, 17, 18, 19, 20, 21, 22^ An additional growing concern is chronic traumatic encephalopathy (CTE). CTE is a neurodegenerative disease that can only be diagnosed postmortem^23^ and has been found in individuals exposed to RHI, particularly former American football players and boxers.^24, 25, 26^ Long-term clinical and neurological consequences related to RHI exposure (including those in CTE), however, are quite heterogeneous and have not been observed in all former American football players.^25, 26, 27, 28, 29^ It is hypothesized that RHI exposure interacts with other risk factors (for example,, genetic and environmental) to alter vulnerability to long-term neurological dysfunction.

Age of first exposure (AFE) to football may be one modifier of later-life neurological and clinical outcomes. Youth football is played between ages of 5 and 14, a period when the brain undergoes substantial maturation in males.^30, 31, 32, 33, 34, 35, 36, 37, 38, 39, 40, 41, 42^ Exposure to RHI over a single season of youth football (without diagnosed concussions) has been associated with white matter alterations in 8–13 year olds.^43^ RHI exposure during peak neurodevelopment may disrupt normal brain maturation to increase vulnerability to long-term clinical impairments, especially in the context of continued football participation.^44, 45^ In a study of former National Football League (NFL) players, subjects who began playing football before age 12 exhibited greater verbal memory and executive dysfunction,^44^ and reduced microstructural integrity of the anterior corpus callosum^45^ in middle age, compared with those who began playing football at 12 or older. These findings were not replicated in a recent NFL-funded study that examined years of youth football play and clinical outcomes in a sample of 45 former NFL players.^46^

The few studies that have reported on AFE to football and long-term clinical function are limited by small sample size, inclusion of only former *professional* football players and lack of assessment of neuropsychiatric features, including behavioral and mood functioning—clinical domains affected by RHI exposure and CTE.^27, 47, 48^ Here, we examined the relationship between AFE to football and behavior, mood and cognitive outcomes in a large cohort of both amateur (that is, those who played only through high school or college) and professional American football players.

## Materials and methods

### Participants

The sample included 214 former American football players from the ongoing Longitudinal Examination to Gather Evidence of Neurodegenerative Disease (LEGEND) study at the Boston University Alzheimer’s Disease (AD) and CTE Center. A description of LEGEND has been provided previously.^1, 5, 49^ LEGEND is a longitudinal research registry of living active and former contact and non-contact sport athletes across the country. An objective of LEGEND is to identify risk factors for the short- and long-term consequences of RHI exposure. Participants for LEGEND are recruited through website postings and word of mouth. Inclusion criteria are broad to optimize generalizability and include ⩾18 years of age and history of participation in an organized sport. The present sample included only male former American football players who played high school, college or professional football, and did not participate in any other organized contact sports. Participants with a self-reported history of concussion within 1 year of their initial LEGEND interview were excluded. These inclusion/exclusion criteria were applied to facilitate internal and external validity between AFE to football and clinical outcomes. In addition, previous work from our team examined AFE to football and cognition in former NFL players^44^ who were part of the recently concluded study, ‘Diagnosing and Evaluating Traumatic Encephalopathy using Clinical Tests’ (DETECT). Three DETECT participants who enrolled into LEGEND were excluded to derive an entirely distinct sample of former American football players.

LEGEND study procedures involve annual completion of online self-reported measures of executive function, behavior, mood and a telephone-based objective cognitive assessment. Telephone-administered structured questionnaires ascertain demographic (for example, age, race and education), athletic history (for example, AFE to football and seasons of football play), and military, substance use and medical history. The BU Medical Campus Institutional Review Board approved all study procedures, and all participants provided written informed consent.

### Measures

The measures administered for LEGEND^1^ assess clinical domains affected by RHI exposure^1, 2, 3, 4, 5, 6, 8, 9, 10, 11^ and CTE,^27, 47^ and include the Brief Test of Adult Cognition by Telephone (BTACT), Behavior Rating Inventory of Executive Function-Adult Version (BRIEF-A), Center for Epidemiologic Studies Depression Scale (CES-D) and the Apathy Evaluation Scale (AES). The BRIEF-A, CES-D and AES are all completed by subjects online, whereas the BTACT is an objective measure of cognitive function administered by telephone. This study reports on initial LEGEND evaluations. Each measure was examined as a continuous variable, and dichotomized to classify participants as clinically impaired using empirically derived cutoff scores. The measures and cutoffs that reflect clinically meaningful impairment include the following.

#### Brief Test of Adult Cognition by Telephone

The BTACT is a 20-min telephone-based objective assessment of cognition.^50, 51^ Telephone administration of cognitive tests is convenient, inexpensive and validated.^50, 52^ The BTACT evaluates episodic verbal memory, working memory, semantic fluency, task switching, inductive reasoning and processing speed. A global composite score is derived using a bi-factor analytic approach.^50^ Bi-factor global scores are then adjusted for age and gender utilizing a regression-based approach that makes use of data from a healthy normative sample.^53^ Lower scores reflect worse cognition, and impairment was defined as ⩾1.5 s.d.'s below the normative mean.

#### Behavior Rating Inventory of Executive Function-Adult Version

Participants completed an online version of the BRIEF-A,^54, 55^ a validated 75-item self-report instrument that measures executive function behaviors. Participants rate how often executive-related behaviors are problematic using a three-point Likert scale, with higher scores representing worse dysfunction. Two summary indices are derived from the BRIEF-A, the Behavioral Regulation Index (BRI) and the Metacognition Index (MI). Raw scores were converted to *T*-scores using age-adjusted normative data. A higher *T*-score reflects greater dysfunction, with ⩾65 (1.5 s.d. above the normative mean) being clinically impaired.^54, 55^ A BRI *T*-score was not calculated for one participant because of missing raw data.

#### Center for Epidemiologic Studies Depression Scale

The CES-D is a 20-item self-report checklist of depressive symptoms,^56^ and was completed online. The CES-D asks participants to use a four-point Likert scale to rate the presence and severity of depressive symptoms in the past week. Higher scores reflect worse depressive symptomatology, and ⩾16 suggests clinical depression.^57^

#### Apathy Evaluation Scale

The AES is an 18-item self-reported measure of cognitive, behavioral and emotional symptoms of apathy.^58^ The AES was completed online, and participants rated the presence and severity of apathy symptoms in the past 4 weeks using a four-point Likert scale. Higher scores represent greater symptoms of apathy, and ⩾34 defined clinically meaningful apathy.^59^

### Statistical analysis

Analyses were performed with SAS version 9.4 (SAS Institute, Cary, NC, USA). Two-tailed significance tests adjusted for multiple comparisons using the false discovery rate method were conducted with significance set at *P*<0.05. AFE was examined as a dichotomized variable (<12 and ⩾12), and as a continuous variable. Age 12 was targeted based on our previous research,^44, 45^ and the neurodevelopmental literature.^30, 31, 32, 33, 34, 35, 36, 37, 38, 39, 40, 41, 42^ Univariate regressions in this sample further showed that age 12 had superior model fit with clinical measures relative to ages 11 and 13. Multivariate linear mixed-effect models examined the relationship between AFE to football (independent variable) and the clinical measures (BRIEF-A (BRI, MI), BTACT, CES-D and AES) as continuous outcome variables. Mixed-effect models reduce type I error by accounting for correlations between groups, outcomes from the same participant and between same tests. Multivariate logistic regression models estimated by generalized estimating equations (GEE) examined the relationship between AFE and each of the clinical measures dichotomized into clinically impaired or not; GEE was used due to the dichotomous outcomes. All analyses controlled for age, years of education and seasons of football play. Self-reported number of total concussions was not included in the models because this variable is prone to measurement error, as athletes' (including former professional American football players) retrospective recall of concussion history can lack accuracy and reliability.^60, 61^ Seasons of football play is a more reliable estimate and encompass cumulative exposure to both concussion and subconcussive injuries. The sample size for analyses examining the BRIEF-A BRI and the BTACT as dependent variables was reduced to 213 because of missing data on these indices. Because symptoms of depression, apathy, behavioral dysregulation and apathy often co-occur and are related, an important component of the mixed-effect and GEE models is that they account for the intercorrelations among the clinical outcome measures. Thus, the relationship between AFE and each clinical measure is not influenced by its association with the other clinical measures. Because of the use of mixed-effect and GEE models, inclusion of any of the specific clinical tests as a covariate is not necessary, and would also be problematic because of reverse causality, given the bidirectional relationships among the clinical measures.

Two sensitivity analyses were performed. A history of learning disability has been suggested to be a confound of AFE to football and clinical function,^46^ and all models were repeated with history of learning disability included as a covariate. For these sensitivity analyses, the sample size was reduced to 206 because of missing data for history of learning disability; sample size for analyses examining the BRIEF-A BRI and the BTACT as dependent variables was reduced to 205 because of missing data on these indices. We examined whether there was an AFE by level of play interaction on the clinical measures to confirm that findings were not cohort-dependent. Linear mixed-effect and logistic regression models tested whether the cross-product between AFE (both as a dichotomized and continuous variable) and level of play (amateur versus professional) predicted the clinical measures independent of the main effects of AFE and level of play.

## Results

**Figure 1:**
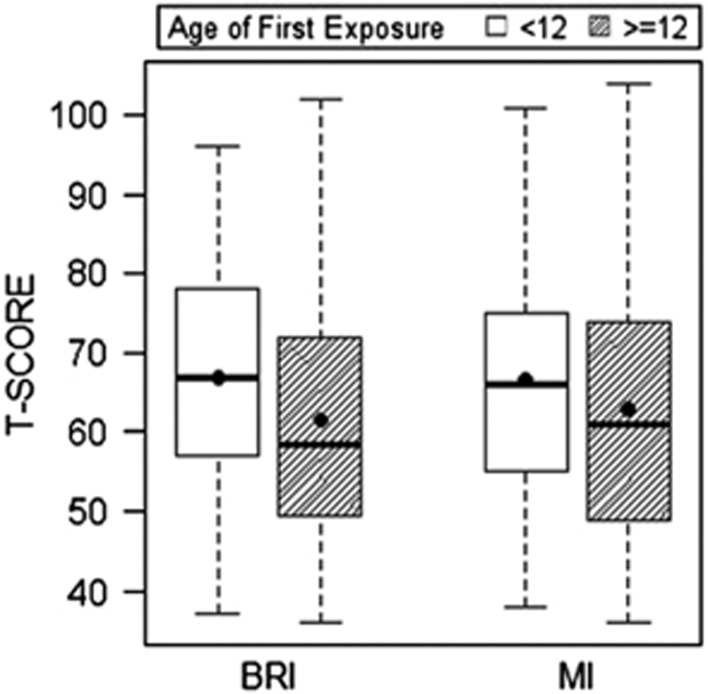
Age of first exposure to American football and reported symptoms of behavioral dysregulation and executive dysfunction in 214 former American football players. Figure presents the results of the linear mixed-effect analyses that showed that those who began playing American football before age 12 exhibited worse (on average) scores on the Behavior Rating Inventory of Executive Function-Adult Version (BRIEF-A) Behavioral Regulation Index (BRI; *P*=0.001) and BRIEF-A Metacognition Index (MI; *P*=0.016). Higher scores represent worse reported clinical function. The circle represents the mean and the horizontal line is the median. The mean group differences were significant after controlling for age, education and total seasons of football play.

**Figure 2:**
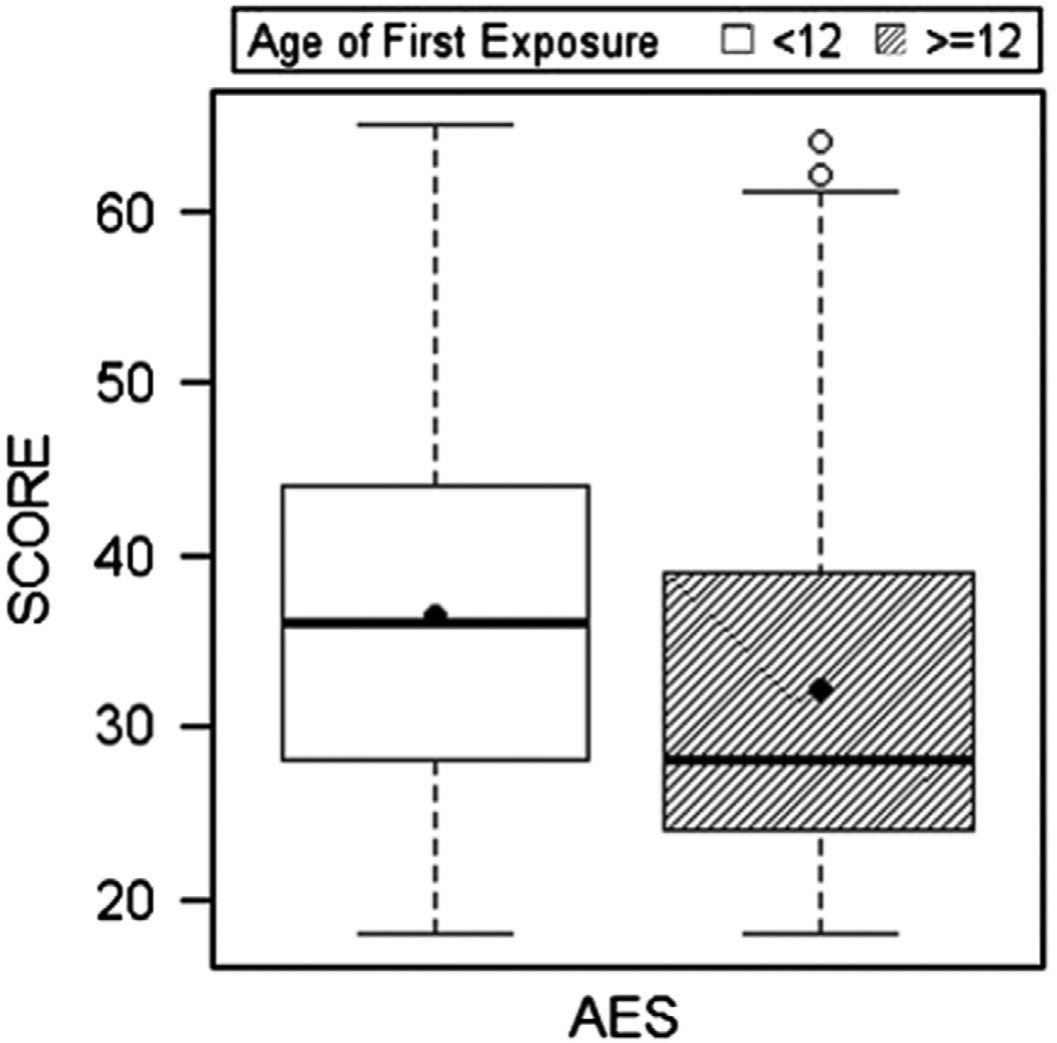
Age of first exposure to American football and reported symptoms of apathy in 214 former American football players. Figure presents the results of the linear mixed-effect analyses that showed that those who began playing American football before age 12 exhibited worse (on average) scores on the Apathy Evaluation Scale (AES), *P*=0.002. Higher scores represent greater reported symptoms of apathy. The circle represents the mean and the horizontal line is the median. The mean group differences were significant after controlling for age, education and total seasons of football play.

**Figure 3:**
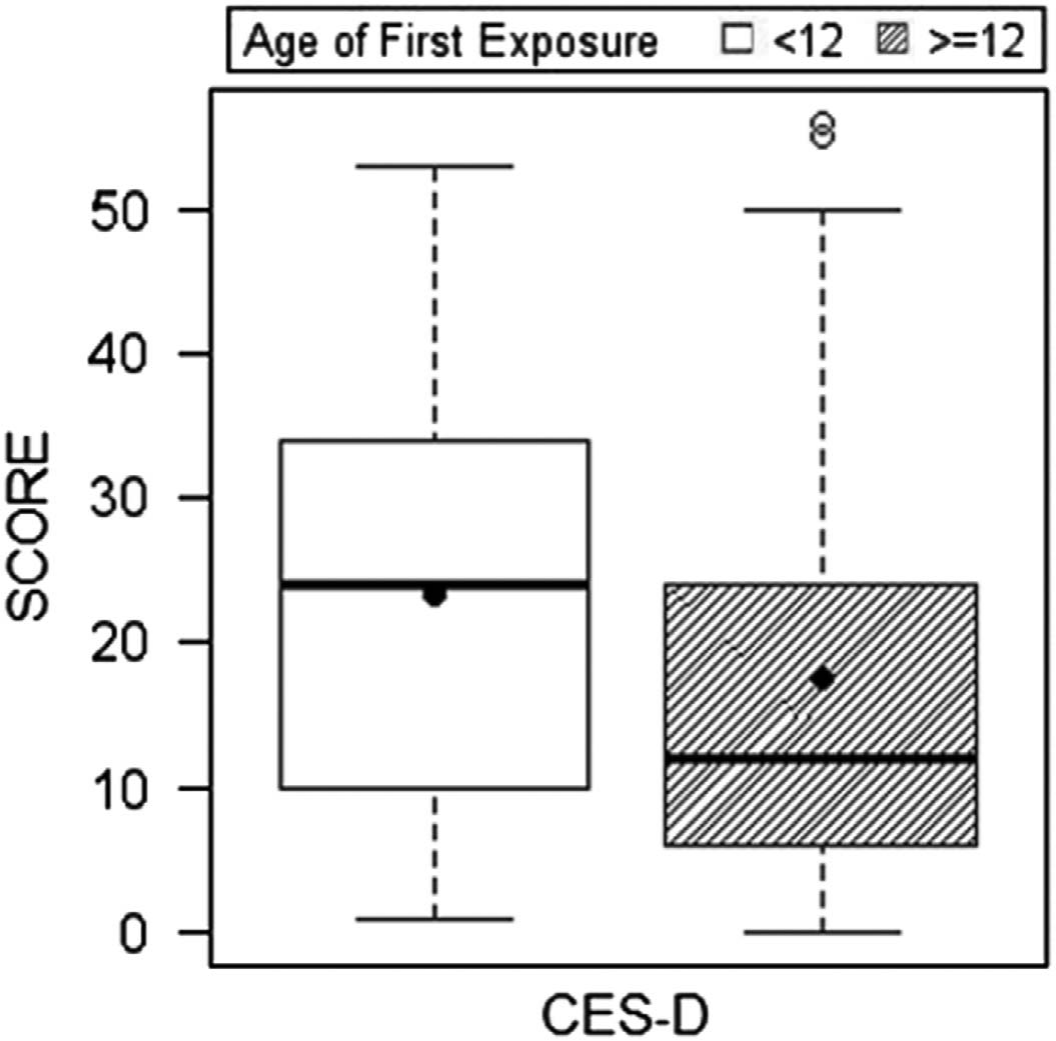
Age of first exposure to American football and reported symptoms of depression in 214 former American football players. Figure presents the results of the linear mixed-effect analyses that showed that those who began playing American football before age 12 exhibited worse (on average) scores on the Center for Epidemiologic Studies Depression Scale (CES-D), *P*=0.001. Higher scores represent greater reported symptoms of depression. The circle represents the mean and the horizontal line is the median. The mean group differences were significant after controlling for age, education and total seasons of football play.

Tables 1 and 2 present sample characteristics. Tables 3 and 4 provide a summary of the mixed-effect and multivariate logistic regression results, including 95% confidence intervals and odds ratios. Among the 214 former football players, compared with those who began playing football ⩾12 years of age, those who started playing <12 exhibited higher (that is, worse) scores on the BRIEF-A (BRI: *P*=0.001; MI: *P*=0.016), CES-D (*P*=0.001) and the AES (*P*=0.002), but not the BTACT (*P*=0.35). See Figures 1, 2, 3. For these measures, those with an AFE to football <12 had >2 × (>3 × for the CES-D) increased odds for clinically meaningful impaired scores, relative to AFE ⩾12: BRIEF-A (BRI: *P*=0.016; MI: *P*=0.016), CES-D (*P*=0.002) and the AES (*P*=0.010). There were no significant effects with BTACT (*P*=0.54). Results remained unchanged when learning disability history was included as a covariate. The AFE by level of play interaction term was not associated with the clinical measures.

When AFE to football was treated as a continuous variable, younger AFE to football was associated with higher (that is, worse) scores on the BRIEF-A (BRI: *P*=0.008; MI: *P*=0.035), CES-D (*P*=0.008) and the AES (*P*=0.008), but not the BTACT (*P*=0.46). Younger AFE corresponded to increased odds for clinically meaningful impaired scores on the CES-D (*P*=0.046) and AES (*P*=0.046). AFE was not significantly associated with clinically elevated scores on the BRIEF-A (BRI: *P*=0.10; MI: *P*=0.09) or the BTACT (*P*=0.62). Learning disability history had minimal influence when included as a covariate; however, there was loss of significance for the BRIEF-A MI (continuous; *P*=0.12) and odds for clinically impaired scores on the CES-D (*P*=0.07). There were no significant associations between the AFE (continuous) by highest level of play interaction term and clinical measures.

## Discussion

In this sample of 214 former American football players, those who began playing football before age 12 had >2 × increased odds for clinically meaningful impairments in reported behavioral regulation, apathy and executive function, and >3 × increased odds for clinically elevated depression scores, compared with those who began playing at 12 or older. Effects were independent of age, education and duration of football play. Younger AFE to football, in general, corresponded with worse behavioral regulation, depression, apathy and executive function, as well as increased odds for clinical depression and apathy. To our knowledge, this study is the first to show a relationship between younger AFE to football and reported clinical dysfunction in a cohort that included both former amateur and professional football players. There was no difference in the effect of AFE by highest level of play. These findings validate and expand upon our previous work in a small, entirely distinct sample of former NFL players,^44^ and extend the influence of AFE to football on clinical function to former football players who only played through high school or college. Overall, this study provides further evidence that playing youth American football may have long-term clinical implications, including behavioral and mood impairments.

A recent study funded by the NFL examined the relationship between years of youth football participation and neuropsychological, neurological and neuroradiological outcomes in 45 former NFL players,^46^ and the authors concluded there were no relationships. That study was limited because a sizable subset of the participants did not play youth football, or only played 1 year. The authors argued that the study by Stamm *et al.* finding significant effects between AFE to football and cognition in former NFL players^44^ was limited by small sample size, decreased generalizability to all football players, arbitrary dichotomization of AFE before and after age 12 and lack of control for a history of learning disability. The present study addresses *all* of these concerns by examining AFE to football as a continuous variable in 214 former American football players, and continues to find a robust relationship between AFE and long-term clinical dysfunction. Inclusion of history of learning disability status as a covariate in the models had minimal influence on the results. Notably, effects were diminished for odds of clinical impairment when AFE was treated as a continuous variable. This could be because the continuous scale assumes a constant effect across all ages; however, there may be a critical age (that is, age 12) where the effects are most robust. Age 12 (an empirically based cutoff) indeed had superior model fit with clinical measures relative to surrounding age cutoffs (that is, ages 11 and 13).

The specific mechanisms underlying the present findings are unknown. Between ages 9 and 12 is a time of peak maturation of gray and white matter volume, synaptic and neurotransmitter densities and glucose utilization, among other neurodevelopmental milestones.^30, 31, 32, 33, 34, 35, 36, 37, 38, 39, 40, 41, 42^ These changes are occurring to structures such as the hippocampus and amygdala,^30, 41^ where the neural circuitry modulates clinical functions, such as emotion regulation and behavior.^63, 64, 65^ Structures like the amygdala have robust connectivity with the ventromedial prefrontal cortex, a region associated with depression in children and young adults.^66^ During this time of peak neurodevelopment, current youth American football players have been estimated, using helmet accelerometry, to experience a median of 252 head impacts per season in one study^67^ and a mean of 240 in another.^68^ RHI exposure over a single season of youth football can result in alterations of the left inferior fronto-occipital fasciculus and right superior longitudinal fasciculus white matter tracts.^43^ Exposure to RHI, in general, is associated with acute and long-term structural, functional and molecular brain changes based on research in active^12, 69, 70, 71, 72^ and former^3, 7, 13, 14, 15, 16, 17, 18, 19, 20, 21, 22^ amateur and/or professional football players. The effects of RHI exposure on the brain at a young age may disrupt neurodevelopment and increase vulnerability to the long-term neuropsychiatric and cognitive impairments associated with prolonged exposure to RHI,^1, 2, 3, 4, 5, 6, 8, 9, 10, 11, 12, 48^ aging or likely both. AFE to football may contribute to why some former American football players develop long-term clinical impairments, whereas others appear more resistant. Some support for this claim can be found in the setting of acute concussion, where children and adolescents are more vulnerable (compared with adults) to prolonged symptoms,^73, 74, 75, 76, 77, 78, 79, 80^ disruptions in educational and social development^76, 81^ and lower intellect and academic achievement.^73, 76^

This is not a study of risk for CTE or of other neurodegenerative disease. CTE currently cannot be diagnosed during life and the presence of CTE in this sample is unknown. Neuropathological evidence of CTE has been documented in former American football players. The current study found that AFE to football was associated with increased odds for impairments in domains described in the literature as being part of the clinical manifestation of CTE,^47^ that is, executive dysfunction, behavioral dysregulation, depression and apathy. These clinical features are not specific to CTE. Young AFE to football may be a risk factor or modifier of the clinical and neuropathological course of CTE, and we are currently conducting clinicopathological investigations in autopsy-confirmed cases of CTE to examine this possibility.^82^

We found no association between AFE to football and cognition as measured by the BTACT. This finding was unexpected, given previous work from our group showed that former NFL players who began playing football before age 12 exhibited worse neuropsychological test performance on measures of episodic memory and executive function at mid-life, compared with those who began playing football at 12 or older.^44^ In the current sample, only 15 (7.0%) participants were clinically impaired on the BTACT, whereas rates of impairments were >40% across all other clinical measures. Higher rates of impairment on non-BTACT measures may be due to their self-report nature, particularly as this convenience sample of self-selected participants may have been more likely to participate because of perceived clinical symptoms. Alternatively, although the BTACT is valid, convenient and cost-effective, telephone assessment of cognition is not ideal and the BTACT is not a comprehensive assessment of cognition. The BTACT may not be sensitive to cognitive impairment in this relatively young sample, and the global score that was used in the present study may not capture the diverse and, at times, subtle deficits associated with RHI exposure. Furthermore, evaluation of neuropsychological and neuropsychiatric function through telephone and online instruments precludes the ability to behaviorally observe the participant, limiting opportunity for the evaluator to monitor concentration and engagement in testing, particularly in the context of symptoms of depression and apathy. That said, the largely normal BTACT scores in the present sample argue against potential confounding from lapses in attention or lack of engagement in testing. Future work should further investigate the relationship between AFE to football and cognition using comprehensive neuropsychological testing.

There are several additional limitations to the present findings. As previously mentioned, this is a convenience sample of self-selected participants and randomization of individuals to groups based on their AFE to football is not possible. A convenience sample could potentially lead to bias effects, especially if AFE plays a role in selection. The findings can only be generalized to male former football players, and the relationship between AFE to other contact sports (for example, soccer) and clinical outcomes, including female contact sports, is unknown and should be the target of future research. There was a wide age range in the sample and older subjects may have had fewer opportunities to participate in organized youth football because it was not widely available until the 1960s. The style of youth football play could have differed across the age groups of the sample, including differences in type and use of protective headgear. We addressed the concern for confound of differences in era of play by including age as a covariate. The causal relationship between AFE to football and long-term clinical outcomes remains unclear, partially because this study was cross-sectional. The cross-sectional study design also limits interpretation of the associations among the clinical outcome measures. The tests examined assess symptoms that often co-occur, with bidirectional relationships (for example, depressive symptoms can affect performance on cognitive tests, cognitive impairment can also lead to symptoms of depression, depressive symptoms and impaired cognition can both be clinical manifestations of a single underlying disorder). Although the mixed-effect and GEE models used accounted for intercorrelations among the clinical measures, it remains challenging to disentangle their exact interactions and subsequent influence on the current findings. In particular, it is unclear whether the reported symptoms are part of a single clinical syndrome, or reflect distinct pathophysiological processes, possibly from the effects of exposure to RHI on strategic brain regions that modulate each of the clinical functions examined. Importantly, although we found significant mean group differences in reported clinical function between the AFE groups, our results underscore that there is significant individual variability in this relationship (Figure 1). Findings from the current study should not be used to inform safety and/or policy decisions in regards to youth football. Any decisions regarding reducing or eliminating youth football must be made with the understanding of the important health and psychosocial benefits of participating in athletics and team sports during pre-adolescence. Future longitudinal studies that objectively monitor the clinical function of youth football players throughout life, including those who do *not* go on to play football at the high school, college or professional level, are ultimately needed to understand the long-term neurological safety implications of youth tackle football.

## Conclusions

Youth exposure to football may have long-term neurobehavioral consequences. Additional research studies, especially large cohort longitudinal studies, are needed to better understand the potential long-term clinical implications of youth American football to inform policy and safety decision-making.

## Figures and Tables

**Table 1:** Sample characteristics

	*Total sample (*N=*214)*	*AFE <12 (*n=*101)*	*AFE* ⩾*12 (*n=*113)*	P
Age, mean (s.d.) years	50.68 (13.33)	48.22 (10.87)	52.87 (14.91)	**0.009**
Race, *n* (%) white	192 (89.7)	92 (91.1)	100 (88.5)	0.53
Education, mean (s.d.) years	17.07 (2.27)	17.09 (2.38)	17.04 (2.19)	0.89
Learning disability (*N*=206 due to missing data), *n* (%) yes	19 (9.2)	10 (10.6)	9 (8.0)	0.52
Reported psychotropic medication, *n* (%) yes	77 (36.0)	39 (38.6)	38 (33.6)	0.45
Reported psychiatric diagnosis, *n* (%) yes (*N*=167 due to missing data)^a^	100 (59.9)	52 (65.8)	48 (54.5)	0.14
Seasons of football play, median (IQR)	12.25 (9)	14.00 (10)	10.00 (8)	**<0.001**
AFE to football, mean (s.d.)	11.12 (2.47)	8.98 (1.65)	13.04 (1.14)	**<0.001**
Total number of concussions (*N*=210 due to missing data), median (IQR)^b^	17.75 (37)	25.00 (88)	15.00 (23)	**<0.001**
Total number of concussions outside of sport/military (*N*=208 due to missing data), median (IQR)^c^	1.00 (2)	1.00 (2)	1.00 (2)	0.17
				
*Highest level of football play*, n *(%)*				0.54
High school	43 (20.1)	20 (19.8)	23 (20.4)	
College	103 (48.1)	51 (50.5)	52 (46.0)	
Professional	68 (31.8)	30 (29.7)	38 (33.6)	
				
*Primary position*, n *(%)*^d^				—
Offensive linemen	69 (32.7)	26 (26.3)	43 (38.4)	
Running back	64 (30.3)	35 (35.4)	29 (25.9)	
Tight end	27 (12.8)	15 (15.2)	12 (10.7)	
Offensive skill	51 (24.2)	23 (23.2)	28 (25.0)	
Defensive line	72 (34.8)	30 (30.3)	42 (38.9)	
Linebacker	58 (28.0)	31 (31.3)	27 (25.0)	
Defensive back	77 (37.2)	38 (38.4)	39 (36.1)	

Abbreviations: AFE, age of first exposure; IQR, interquartile range.

Independent sample *t*-tests and *χ*^2^-analyses were used to compare differences between the AFE groups, except for seasons of football play, total number of concussions and total number of concussions outside of sport/military for which Mann–Whitney *U-*test was conducted due to a non-normal distribution. Proportion of white versus other was tested and the highest level of football play was transformed to amateur versus professional.

a Includes reported history of depression, anxiety, bipolar disorder, schizophrenia and/or other psychiatric difficulties.

b Self-reported number of concussions after being provided with a modern definition of concussion.^62^

c Only 39 (18.2%) subjects reported a military history, and the median number of head injuries experienced during the military was 0 (IQR=1).

d Players could indicate both a primary offensive and defensive position and therefore could be represented more than once. The bold is used for those *P* values that are significant.

**Table 2:** Clinical test performance

*Test*	*Total sample (*N=*214)*		*AFE <12 (*n=*101)*		*AFE* ⩾*12 (*n=*113)*	
	*Mean (s.d.)*	n *(%), Impaired*	*Mean (s.d.)*	n *(%), Impaired*	*Mean (s.d.)*	n *(%), Impaired*
BRIEF-A BRI^a^	64.11 (15.16)	94 (44.1)	67.01 (14.79)	51 (50.5)	61.50 (15.07)	43 (38.4)
BRIEF-A MI	64.71 (15.54)	103 (48.1)	66.66 (14.73)	55 (54.5)	62.97 (16.09)	48 (42.5)
BTACT^a^	−0.20 (0.90)	15 (7.0)	−0.31 (0.87)	8 (7.9)	−0.11 (0.92)	7 (6.3)
AES	34.15 (11.05)	97 (45.3)	36.42 (10.85)	56 (55.4)	32.12 (10.88)	41 (36.3)
CES-D	20.24 (14.48)	117 (54.7)	23.25 (13.85)	67 (66.3)	17.55 (14.57)	50 (44.2)

Abbreviations: AES, Apathy Evaluation Scale; AFE, age of first exposure; BRI, Behavioral Regulation Index; BRIEF-A, Behavior Rating Inventory of Executive Function-Adult Version; BTACT, Brief Test of Adult Cognition by Telephone; CES-D, Center for Epidemiologic Studies Depression Scale; MI, Metacognition Index.

% impaired includes those who scored above empirically derived cutoff scores that reflect clinical impairment, which includes: CES-D⩾16, AES⩾34, BRIEF-A BRI and MI⩾65, and BTACT⩽−1.5. BRIEF-A subtests are *T*-scores and BTACT are age- and gender-adjusted bi-factor scores.

a *N*=213 in the overall sample because of missing data.

**Table 3:** Summary of linear mixed-effects models examining AFE to football and behavior, mood and cognitive function

*214 Former American football players*								
*Clinical tests*	*AFE dichotomized (1, <12; 0*, ⩾*12)*				*AFE continuous*			
	*Est*	*95% CI*	P	*Adj*. P^a^	*Est*	*95% CI*	P	*Adj*. P^a^
BRIEF-A BRI	7.65	3.54, 11.76	**<0.001**	**0.001**	−1.35	−2.20, −0.50	**0.002**	**0.008**
BRIEF-A MI	5.50	1.19, 9.80	**0.013**	**0.016**	−0.99	−1.88, −0.11	**0.028**	**0.035**
BTACT	−12.26	−38.18, 13.65	0.35	0.35	2.00	−3.33, 7.34	0.46	0.46
AES	5.10	2.04, 8.15	**0.001**	**0.002**	−0.93	−1.56, −0.30	**0.004**	**0.008**
CES-D	7.29	3.38, 11.21	**<0.001**	**0.001**	−1.17	−1.98, −0.36	**0.005**	**0.008**

Abbreviations: Adj, adjusted; AES, Apathy Evaluation Scale; AFE, age of first exposure; BRI, Behavioral Regulation Index; BRIEF-A, Behavior Rating Inventory of Executive Function-Adult Version; BTACT, Brief Test of Adult Cognition by Telephone; CES-D, Center for Epidemiologic Studies Depression Scale; CI, confidence interval; Est, estimate; MI, Metacognition Index.

All clinical tests were examined as continuous variables. Lower scores on the BTACT and higher scores on the BRIEF-A subtests, AES and CES-D reflect worse clinical function. Analyses adjusted for age, years of education and seasons of football play. A factor of 100 was applied to the BTACT to facilitate model fit.

a *P*-values are adjusted for multiple comparisons via the false discovery rate method. The bold is used for those *P* values that are significant.

**Table 4:** Summary of multivariate logistic regression models examining AFE to football and clinically meaningful scores on measures of behavior, mood and cognitive function

*214 Former American football players*								
	*AFE dichotomized (1, <12; 0*, ⩾*12)*				*Continuous AFE*			
*Clinical tests*	*OR*	*95% CI*	P	*Adj*. P^a^	*OR*	*95% CI*	P	*Adj*. P^a^
BRIEF-A BRI	2.16	1.19, 3.91	**0.011**	**0.016**	0.89	0.79, 1.01	0.08	0.10
BRIEF-A MI	2.10	1.17, 3.76	**0.013**	**0.016**	0.89	0.78, 1.00	0.05	0.09
BTACT	1.43	0.46, 4.41	0.54	0.54	0.94	0.75, 1.18	0.62	0.62
AES	2.39	1.32, 4.32	**0.004**	**0.010**	0.86	0.76, 0.97	**0.014**	**0.046**
CES-D	3.08	1.65, 5.76	**<0.001**	**0.002**	0.85	0.74, 0.97	**0.018**	**0.046**

Abbreviations: Adj, adjusted; AES, Apathy Evaluation Scale; AFE, age of first exposure; BRI, Behavioral Regulation Index; BRIEF-A, Behavior Rating Inventory of Executive Function-Adult Version; BTACT, Brief Test of Adult Cognition by Telephone; CES-D, Center for Epidemiologic Studies Depression Scale; CI, confidence interval; MI, Metacognition Index; OR, odds ratio.

Clinical tests were dichotomized into impaired or not impaired using established cutoffs; higher scores reflect clinical impairment. Analyses adjusted for age, years of education and seasons of football play. A factor of 100 was applied to the BTACT to facilitate model fit.

a *P*-values are adjusted for multiple comparisons via the false discovery rate method. The bold is used for those *P* values that are significant.

## References

[bib1] Montenigro PH, Alosco ML, Martin BM, Daneshvar DH, Mez J, Chaisson CE et al. Cumulative head impact exposure predicts later-life depression, apathy, executive dysfunction, and cognitive impairment in former high school and college football players. J Neurotrauma 2016; 34: 328–340. 27029716 10.1089/neu.2016.4413 PMC5220530

[bib2] Alosco ML, Jarnagin J, Tripodis Y, Platt M, Martin B, Chaisson CE et al. Olfactory function and associated clinical correlates in former national football league players. J Neurotrauma 2016; 34: 772–780. 27430424 10.1089/neu.2016.4536 PMC5314992

[bib3] Gardner RC, Possin KL, Hess CP, Huang EJ, Grinberg LT, Nolan AL et al. Evaluating and treating neurobehavioral symptoms in professional American football players: lessons from a case series. Neurol Clin Pract 2015; 5: 285–295. 26336629 10.1212/CPJ.0000000000000157 PMC4549717

[bib4] Randolph C, Karantzoulis S, Guskiewicz K. Prevalence and characterization of mild cognitive impairment in retired national football league players. J Int Neuropsychol Soc 2013; 19: 873–880. 23902607 10.1017/S1355617713000805

[bib5] Seichepine DR, Stamm JM, Daneshvar DH, Riley DO, Baugh CM, Gavett BE et al. Profile of self-reported problems with executive functioning in college and professional football players. J Neurotrauma 2013; 30: 1299–1304. 23421745 10.1089/neu.2012.2690 PMC3713446

[bib6] Didehbani N, Munro Cullum C, Mansinghani S, Conover H, Hart J Jr. Depressive symptoms and concussions in aging retired NFL players. Arch Clin Neuropsychol 2013; 28: 418–424. 23644673 10.1093/arclin/act028 PMC4007104

[bib7] Hart J Jr, Kraut MA, Womack KB, Strain J, Didehbani N, Bartz E et al. Neuroimaging of cognitive dysfunction and depression in aging retired National Football League players: a cross-sectional study. JAMA Neurol 2013; 70: 326–335. 23303193 10.1001/2013.jamaneurol.340 PMC4016798

[bib8] Guskiewicz KM, Marshall SW, Bailes J, McCrea M, Cantu RC, Randolph C et al. Association between recurrent concussion and late-life cognitive impairment in retired professional football players. Neurosurgery 2005; 57: 719–726, discussion -26. 16239884 10.1093/neurosurgery/57.4.719

[bib9] Guskiewicz KM, Marshall SW, Bailes J, McCrea M, Harding HP Jr, Matthews A et al. Recurrent concussion and risk of depression in retired professional football players. Med Sci Sports Exerc 2007; 39: 903–909. 17545878 10.1249/mss.0b013e3180383da5

[bib10] Kerr ZY, Marshall SW, Harding HP Jr, Guskiewicz KM. Nine-year risk of depression diagnosis increases with increasing self-reported concussions in retired professional football players. Am J Sports Med 2012; 40: 2206–2212. 22922518 10.1177/0363546512456193

[bib11] Wright MJ, Woo E, Birath JB, Siders CA, Kelly DF, Wang C et al. An index predictive of cognitive outcome in retired professional American Football players with a history of sports concussion. J Clin Exp Neuropsychol 2016; 38: 561–571. 26898803 10.1080/13803395.2016.1139057

[bib12] Singh R, Meier TB, Kuplicki R, Savitz J, Mukai I, Cavanagh L et al. Relationship of collegiate football experience and concussion with hippocampal volume and cognitive outcomes. JAMA 2014; 311: 1883–1888. 24825643 10.1001/jama.2014.3313

[bib13] Adler CM, DelBello MP, Weber W, Williams M, Duran LR, Fleck D et al. MRI Evidence of Neuropathic Changes in Former College Football Players. Clin J Sport Med; e-pub ahead of print, 17 October 2016. 10.1097/JSM.0000000000000391 27755011

[bib14] Amen DG, Willeumier K, Omalu B, Newberg A, Raghavendra C, Raji CA. Perfusion neuroimaging abnormalities alone distinguish National Football League players from a healthy population. J Alzheimers Dis 2016; 53: 237–241. 27128374 10.3233/JAD-160207 PMC4942725

[bib15] Gardner RC, Hess CP, Brus-Ramer M, Possin KL, Cohn-Sheehy BI, Kramer JH et al. Cavum septum pellucidum in retired American pro-football players. J Neurotrauma 2016; 33: 157–161. 25970145 10.1089/neu.2014.3805 PMC4696427

[bib16] Hampshire A, MacDonald A, Owen AM. Hypoconnectivity and hyperfrontality in retired American football players. Sci Rep 2013; 3: 2972. 24135857 10.1038/srep02972 PMC6505675

[bib17] Koerte IK, Hufschmidt J, Muehlmann M, Tripodis Y, Stamm JM, Pasternak O et al. Cavum septi pellucidi in symptomatic former professional football players. J Neurotrauma 2016; 33: 346–353. 26414478 10.1089/neu.2015.3880 PMC4761807

[bib18] Lin AP, Ramadan S, Stern RA, Box HC, Nowinski CJ, Ross BD et al. Changes in the neurochemistry of athletes with repetitive brain trauma: preliminary results using localized correlated spectroscopy. Alzheimers Res Ther 2015; 7: 13. 25780390 10.1186/s13195-015-0094-5 PMC4361214

[bib19] Strain JF, Didehbani N, Spence J, Conover H, Bartz EK, Mansinghani S et al. White matter changes and confrontation naming in retired aging National Football League athletes. J Neurotrauma 2016; 34: 372–379. 27297660 10.1089/neu.2016.4446 PMC5220576

[bib20] Strain JF, Womack KB, Didehbani N, Spence JS, Conover H, Hart J Jr et al. Imaging correlates of memory and concussion history in retired National Football League athletes. JAMA Neurol 2015; 72: 773–780. 25985094 10.1001/jamaneurol.2015.0206 PMC6761828

[bib21] Coughlin JM, Wang Y, Minn I, Bienko N, Ambinder EB, Xu X et al. Imaging of glial cell activation and white matter integrity in brains of active and recently retired National Football League players. JAMA Neurol 2017; 74: 67–74. 27893897 10.1001/jamaneurol.2016.3764 PMC5504689

[bib22] Ford JH, Giovanello KS, Guskiewicz KM. Episodic memory in former professional football players with a history of concussion: an event-related functional neuroimaging study. J Neurotrauma 2013; 30: 1683–1701. 23679098 10.1089/neu.2012.2535 PMC3840476

[bib23] McKee AC, Cairns NJ, Dickson DW, Folkerth RD, Keene CD, Litvan I et al. The first NINDS/NIBIB consensus meeting to define neuropathological criteria for the diagnosis of chronic traumatic encephalopathy. Acta Neuropathol 2016; 131: 75–86. 26667418 10.1007/s00401-015-1515-z PMC4698281

[bib24] Ling H, Morris HR, Neal JW, Lees AJ, Hardy J, Holton JL et al. Mixed pathologies including chronic traumatic encephalopathy account for dementia in retired association football (soccer) players. Acta Neuropathol 2017; 133: 337–352. 28205009 10.1007/s00401-017-1680-3 PMC5325836

[bib25] Bieniek KF, Ross OA, Cormier KA, Walton RL, Soto-Ortolaza A, Johnston AE et al. Chronic traumatic encephalopathy pathology in a neurodegenerative disorders brain bank. Acta Neuropathol 2015; 130: 877–889. 26518018 10.1007/s00401-015-1502-4 PMC4655127

[bib26] McKee AC, Stern RA, Nowinski CJ, Stein TD, Alvarez VE, Daneshvar DH et al. The spectrum of disease in chronic traumatic encephalopathy. Brain 2013; 136: 43–64. 23208308 10.1093/brain/aws307 PMC3624697

[bib27] Stern RA, Daneshvar DH, Baugh CM, Seichepine DR, Montenigro PH, Riley DO et al. Clinical presentation of chronic traumatic encephalopathy. Neurology 2013; 81: 1122–1129. 23966253 10.1212/WNL.0b013e3182a55f7f PMC3795597

[bib28] Alosco ML, Mez J, Kowall NW, Stein TD, Goldstein LE, Cantu RC et al. Cognitive reserve as a modifier of clinical expression in chronic traumatic encephalopathy: a preliminary examination. J Neuropsychiatry Clin Neurosci 2016; 29: 6–12. 27539377 10.1176/appi.neuropsych.16030043 PMC5288278

[bib29] Casson IR, Viano DC, Haacke EM, Kou Z, LeStrange DG. Is there chronic brain damage in retired NFL players? Neuroradiology, neuropsychology, and neurology examinations of 45 retired players. Sports Health 2014; 6: 384–395. 25177413 10.1177/1941738114540270 PMC4137679

[bib30] Caviness VS Jr, Kennedy DN, Richelme C, Rademacher J, Filipek PA. The human brain age 7-11 years: a volumetric analysis based on magnetic resonance images. Cereb Cortex 1996; 6: 726–736. 8921207 10.1093/cercor/6.5.726

[bib31] Chugani HT, Phelps ME, Mazziotta JC. Positron emission tomography study of human brain functional development. Ann Neurol 1987; 22: 487–497. 3501693 10.1002/ana.410220408

[bib32] Courchesne E, Chisum HJ, Townsend J, Cowles A, Covington J, Egaas B et al. Normal brain development and aging: quantitative analysis at *in vivo* MR imaging in healthy volunteers. Radiology 2000; 216: 672–682. 10966694 10.1148/radiology.216.3.r00au37672

[bib33] Epstein HT. Stages of increased cerebral blood flow accompany stages of rapid brain growth. Brain Dev 1999; 21: 535–539. 10598054 10.1016/s0387-7604(99)00066-2

[bib34] Giedd JN. The teen brain: insights from neuroimaging. J Adolesc Health 2008; 42: 335–343. 18346658 10.1016/j.jadohealth.2008.01.007

[bib35] Giedd JN, Blumenthal J, Jeffries NO, Castellanos FX, Liu H, Zijdenbos A et al. Brain development during childhood and adolescence: a longitudinal MRI study. Nat Neurosci 1999; 2: 861–863. 10491603 10.1038/13158

[bib36] Lebel C, Walker L, Leemans A, Phillips L, Beaulieu C. Microstructural maturation of the human brain from childhood to adulthood. Neuroimage 2008; 40: 1044–1055. 18295509 10.1016/j.neuroimage.2007.12.053

[bib37] Thatcher RW. Maturation of the human frontal lobes. Physiological evidence for staging. Dev Neuropsychol 1991; 7: 397–419.

[bib38] Shaw P, Greenstein D, Lerch J, Clasen L, Lenroot R, Gogtay N et al. Intellectual ability and cortical development in children and adolescents. Nature 2006; 440: 676–679. 16572172 10.1038/nature04513

[bib39] Shaw P, Kabani NJ, Lerch JP, Eckstrand K, Lenroot R, Gogtay N et al. Neurodevelopmental trajectories of the human cerebral cortex. J Neurosci 2008; 28: 3586–3594. 18385317 10.1523/JNEUROSCI.5309-07.2008 PMC6671079

[bib40] Snook L, Paulson LA, Roy D, Phillips L, Beaulieu C. Diffusion tensor imaging of neurodevelopment in children and young adults. Neuroimage 2005; 26: 1164–1173. 15961051 10.1016/j.neuroimage.2005.03.016

[bib41] Uematsu A, Matsui M, Tanaka C, Takahashi T, Noguchi K, Suzuki M et al. Developmental trajectories of amygdala and hippocampus from infancy to early adulthood in healthy individuals. PLoS ONE 2012; 7: e46970. 23056545 10.1371/journal.pone.0046970 PMC3467280

[bib42] Gogtay N, Giedd JN, Lusk L, Hayashi KM, Greenstein D, Vaituzis AC et al. Dynamic mapping of human cortical development during childhood through early adulthood. Proc Natl Acad Sci USA 2004; 101: 8174–8179. 15148381 10.1073/pnas.0402680101 PMC419576

[bib43] Bahrami N, Sharma D, Rosenthal S, Davenport EM, Urban JE, Wagner B et al. Subconcussive head impact exposure and white matter tract changes over a single season of youth football. Radiology 2016; 281: 919–926. 27775478 10.1148/radiol.2016160564 PMC5131834

[bib44] Stamm JM, Bourlas AP, Baugh CM, Fritts NG, Daneshvar DH, Martin BM et al. Age of first exposure to football and later-life cognitive impairment in former NFL players. Neurology 2015; 84: 1114–1120. 25632088 10.1212/WNL.0000000000001358 PMC4371403

[bib45] Stamm JM, Koerte IK, Muehlmann M, Pasternak O, Bourlas AP, Baugh CM et al. Age at first exposure to football is associated with altered corpus callosum white matter microstructure in former professional football players. J Neurotrauma 2015; 32: 1768–1776. 26200068 10.1089/neu.2014.3822 PMC4651044

[bib46] Solomon GS, Kuhn AW, Zuckerman SL, Casson IR, Viano DC, Lovell MR et al. Participation in pre-high school football and neurological, neuroradiological, and neuropsychological findings in later life: a study of 45 retired National Football League players. Am J Sports Med 2016; 44: 1106–1115. 26888877 10.1177/0363546515626164

[bib47] Montenigro PH, Baugh CM, Daneshvar DH, Mez J, Budson AE, Au R et al. Clinical subtypes of chronic traumatic encephalopathy: literature review and proposed research diagnostic criteria for traumatic encephalopathy syndrome. Alzheimers Res Ther 2014; 6: 68. 25580160 10.1186/s13195-014-0068-z PMC4288217

[bib48] Bryan CJ, Clemans TA. Repetitive traumatic brain injury, psychological symptoms, and suicide risk in a clinical sample of deployed military personnel. JAMA Psychiatry 2013; 70: 686–691. 23676987 10.1001/jamapsychiatry.2013.1093

[bib49] Robbins CA, Daneshvar DH, Picano JD, Gavett BE, Baugh CM, Riley DO et al. Self-reported concussion history: impact of providing a definition of concussion. Open Access J Sports Med 2014; 5: 99–103. 24891816 10.2147/OAJSM.S58005 PMC4019619

[bib50] Gavett BE, Crane PK, Dams-O'Connor K. Bi-factor analyses of the Brief Test of Adult Cognition by Telephone. NeuroRehabilitation 2013; 32: 253–265. 23535786 10.3233/NRE-130842 PMC4489934

[bib51] Tun PA, Lachman ME. Telephone assessment of cognitive function in adulthood: the Brief Test of Adult Cognition by Telephone. Age Ageing 2006; 35: 629–632. 16943264 10.1093/ageing/afl095

[bib52] Wilson RS, Bennett DA. Assessment of cognitive decline in old age with brief tests amenable to telephone administration. Neuroepidemiology 2005; 25: 19–25. 15855801 10.1159/000085309

[bib53] Gurnani AS, John SE, Gavett BE. Regression-based norms for a bi-factor model for scoring the Brief Test of Adult Cognition by Telephone (BTACT). Arch Clin Neuropsychol 2015; 30: 280–291. 25724515 10.1093/arclin/acv005 PMC4635635

[bib54] Roth RM, Isquith PK, Gioia GA. BRIEF-A: Behavior Rating Inventory of Executive Function-Adult Version: Professional Manual. Psychological Assessment Resources: Lutz, FL, USA, 2005.

[bib55] Gioia GA, Isquith PK, Guy SC, Kenworthy L. Behavior rating inventory of executive function. Child Neuropsychol 2000; 6: 235–238. 11419452 10.1076/chin.6.3.235.3152

[bib56] Radloff LS. The CES-D scale: a self-report depression scale for research in the general population. Appl Psychol Meas 1977; 1: 385–401.

[bib57] Lewinsohn PM, Seeley JR, Roberts RE, Allen NB. Center for Epidemiologic Studies Depression Scale (CES-D) as a screening instrument for depression among community-residing older adults. Psychol Aging 1997; 12: 277–287. 9189988 10.1037//0882-7974.12.2.277

[bib58] Marin RS, Biedrzycki RC, Firinciogullari S. Reliability and validity of the Apathy Evaluation Scale. Psychiatry Res 1991; 38: 143–162. 1754629 10.1016/0165-1781(91)90040-v

[bib59] Kant R, Duffy JD, Pivovarnik A. Prevalence of apathy following head injury. Brain Inj 1998; 12: 87–92. 9483342 10.1080/026990598122908

[bib60] Kerr ZY, Marshall SW, Guskiewicz KM. Reliability of concussion history in former professional football players. Med Sci Sports Exerc 2012; 44: 377–382. 21857370 10.1249/MSS.0b013e31823240f2

[bib61] Kerr ZY, Mihalik JP, Guskiewicz KM, Rosamond WD, Evenson KR, Marshall SW. Agreement between athlete-recalled and clinically documented concussion histories in former collegiate athletes. Am J Sports Med 2015; 43: 606–613. 25560539 10.1177/0363546514562180

[bib62] Alosco ML, Jarnagin J, Tripodis Y, Martin B, Chaisson C, Baugh CM et al. Utility of providing a concussion definition in the assessment of concussion history in former NFL players. Brain Inj 2017; 31: 1116–1123. 28471243 10.1080/02699052.2017.1294709 PMC6157273

[bib63] Gallagher M, Chiba AA. The amygdala and emotion. Curr Opin Neurobiol 1996; 6: 221–227. 8725964 10.1016/s0959-4388(96)80076-6

[bib64] Rasia-Filho AA, Londero RG, Achaval M. Functional activities of the amygdala: an overview. J Psychiatry Neurosci 2000; 25: 14–23. 10721680 PMC1407702

[bib65] Phelps EA. Human emotion and memory: interactions of the amygdala and hippocampal complex. Curr Opin Neurobiol 2004; 14: 198–202. 15082325 10.1016/j.conb.2004.03.015

[bib66] Ducharme S, Albaugh MD, Hudziak JJ, Botteron KN, Nguyen TV, Truong C et al. Anxious/depressed symptoms are linked to right ventromedial prefrontal cortical thickness maturation in healthy children and young adults. Cereb Cortex 2014; 24: 2941–2950. 23749874 10.1093/cercor/bht151 PMC4193463

[bib67] Munce TA, Dorman JC, Thompson PA, Valentine VD, Bergeron MF. Head impact exposure and neurologic function of youth football players. Med Sci Sports Exerc 2015; 47: 1567–1576. 25437194 10.1249/MSS.0000000000000591

[bib68] Cobb BR, Urban JE, Davenport EM, Rowson S, Duma SM, Maldjian JA et al. Head impact exposure in youth football: elementary school ages 9-12 years and the effect of practice structure. Ann Biomed Eng 2013; 41: 2463–2473. 23881111 10.1007/s10439-013-0867-6 PMC3825505

[bib69] Mayinger MC, Merchant-Borna K, Hufschmidt J, Muehlmann M, Weir IR, Rauchmann BS et al. White matter alterations in college football players: a longitudinal diffusion tensor imaging study. Brain Imaging Behav; e-pub ahead of print 14 January 2017. 10.1007/s11682-017-9672-4 28092023

[bib70] Bazarian JJ, Zhu T, Zhong J, Janigro D, Rozen E, Roberts A et al. Persistent, long-term cerebral white matter changes after sports-related repetitive head impacts. PLoS ONE 2014; 9: e94734. 24740265 10.1371/journal.pone.0094734 PMC3989251

[bib71] Abbas K, Shenk TE, Poole VN, Robinson ME, Leverenz LJ, Nauman EA et al. Effects of repetitive sub-concussive brain injury on the functional connectivity of Default Mode Network in high school football athletes. Dev Neuropsychol 2015; 40: 51–56. 25649781 10.1080/87565641.2014.990455

[bib72] Poole VN, Breedlove EL, Shenk TE, Abbas K, Robinson ME, Leverenz LJ et al. Sub-concussive hit characteristics predict deviant brain metabolism in football athletes. Dev Neuropsychol 2015; 40: 12–17. 25649774 10.1080/87565641.2014.984810

[bib73] Anderson V, Spencer-Smith M, Wood A. Do children really recover better? Neurobehavioural plasticity after early brain insult. Brain 2011; 134: 2197–2221. 21784775 10.1093/brain/awr103

[bib74] Guskiewicz KM, Valovich McLeod TC. Pediatric sports-related concussion. PM R 2011; 3: 353–364, quiz 64. 21497322 10.1016/j.pmrj.2010.12.006

[bib75] Lovell MR, Collins MW, Iverson GL, Johnston KM, Bradley JP. Grade 1 or "ding" concussions in high school athletes. Am J Sports Med 2004; 32: 47–54. 14754723 10.1177/0363546503260723

[bib76] Moser RS, Schatz P, Jordan BD. Prolonged effects of concussion in high school athletes. Neurosurgery 2005; 57: 300–306, discussion-6. 16094159 10.1227/01.neu.0000166663.98616.e4

[bib77] Sim A, Terryberry-Spohr L, Wilson KR. Prolonged recovery of memory functioning after mild traumatic brain injury in adolescent athletes. J Neurosurg 2008; 108: 511–516. 18312098 10.3171/JNS/2008/108/3/0511

[bib78] Covassin T, Elbin RJ, Harris W, Parker T, Kontos A. The role of age and sex in symptoms, neurocognitive performance, and postural stability in athletes after concussion. Am J Sports Med 2012; 40: 1303–1312. 22539534 10.1177/0363546512444554

[bib79] Field M, Collins MW, Lovell MR, Maroon J. Does age play a role in recovery from sports-related concussion? A comparison of high school and collegiate athletes. J Pediatr 2003; 142: 546–553. 12756388 10.1067/mpd.2003.190

[bib80] Zuckerman SL, Lee YM, Odom MJ, Solomon GS, Forbes JA, Sills AK. Recovery from sports-related concussion: days to return to neurocognitive baseline in adolescents versus young adults. Surg Neurol Int 2012; 3: 130. 23227435 10.4103/2152-7806.102945 PMC3513851

[bib81] Gioia GA, Schneider JC, Vaughan CG, Isquith PK. Which symptom assessments and approaches are uniquely appropriate for paediatric concussion? Br J Sports Med 2009; 43(Suppl 1): i13–i22. 19433419 10.1136/bjsm.2009.058255

[bib82] Mez J, Solomon TM, Daneshvar DH, Murphy L, Kiernan PT, Montenigro PH et al. Assessing clinicopathological correlation in chronic traumatic encephalopathy: rationale and methods for the UNITE study. Alzheimers Res Ther 2015; 7: 62. 26455775 10.1186/s13195-015-0148-8 PMC4601147

